# Characterization of the protease domain of Rice tungro bacilliform virus responsible for the processing of the capsid protein from the polyprotein

**DOI:** 10.1186/1743-422X-2-33

**Published:** 2005-04-14

**Authors:** Philippe Marmey, Ana Rojas-Mendoza, Alexandre de Kochko, Roger N Beachy, Claude M Fauquet

**Affiliations:** 1 IRD, UMR «DGPC», B.P. 64501, 34394 Montpellier cedex 5, France; 2 Protein Design Group, Centro Nacional de Biotecnologia, Campus Universidad Autonoma Cantoblanco, 28049 Madrid, Spain; 3 Donald Danforth Plant Science Center, 975 North Warson Road, St. Louis, MO 63132, USA

## Abstract

**Background:**

Rice tungro bacilliform virus (RTBV) is a pararetrovirus, and a member of the family *Caulimoviridae *in the genus *Badnavirus*. RTBV has a long open reading frame that encodes a large polyprotein (P3). Pararetroviruses show similarities with retroviruses in molecular organization and replication. P3 contains a putative movement protein (MP), the capsid protein (CP), the aspartate protease (PR) and the reverse transcriptase (RT) with a ribonuclease H activity. PR is a member of the cluster of retroviral proteases and serves to proteolytically process P3. Previous work established the N- and C-terminal amino acid sequences of CP and RT, processing of RT by PR, and estimated the molecular mass of PR by western blot assays.

**Results:**

A molecular mass of a protein that was associated with virions was determined by in-line HPLC electrospray ionization mass spectral analysis. Comparison with retroviral proteases amino acid sequences allowed the characterization of a putative protease domain in this protein. Structural modelling revealed strong resemblance with retroviral proteases, with overall folds surrounding the active site being well conserved. Expression in *E. coli *of putative domain was affected by the presence or absence of the active site in the construct. Analysis of processing of CP by PR, using pulse chase labelling experiments, demonstrated that the 37 kDa capsid protein was dependent on the presence of the protease in the constructs.

**Conclusion:**

The findings suggest the characterization of the RTBV protease domain. Sequence analysis, structural modelling, *in vitro *expression studies are evidence to consider the putative domain as being the protease domain. Analysis of expression of different peptides corresponding to various domains of P3 suggests a processing of CP by PR. This work clarifies the organization of the RTBV polyprotein, and its processing by the RTBV protease.

## Background

Plant pararetroviruses are classified as members of the family *Caulimoviridae *which comprises 6 genera [1]. Like retroviruses, members of this group of viruses use reverse transcriptase for replication of the genome [2,3]. However, they differ in two major points: retroviruses have an RNA genome whereas pararetroviruses have a DNA genome; and, a proviral form of retroviruses is integrated into the host chromosome whereas the DNA of pararetroviruses accumulates within the nucleus as multiple copies of a circular chromosome [4].

Retroviruses and pararetroviruses show similarities in their molecular organization and replication process and are phylogenetically related. These groups of viruses direct the production of a terminally redundant RNA which is used as a replicative intermediate and as mRNA. Many of the genes encoded by pararetroviruses are homologous in sequence and/or analogous in function to those of retroviruses. The genome of all replication-competent retroviruses consists of three major genetic elements that are arranged in the order gag-pol-env (structural-replication-envelope proteins). Each protein is produced as a result of frameshifting during translation, or suppression of stop codons in the polyproteins. Products of the gag ORF represent the structural components of the viral matrix, i.e. capsid and nucleocapsidproteins; the pol domains generally comprise the protease, reverse transcriptase, ribonuclease and an endonuclease/integrase. Pararetroviruses encode the gag-pol core, but lack an integrase, as viral DNA integration into the host chromosome is not required [5].

Retrovirus and pararetrovirus polyproteins are believed to be processed by their own aspartate proteases [3,6,7]. These proteases contain several conserved regions when compared with each other and share consensus sequences in the active site; however, they show no homology with other viral proteases. Protein cleavages by these and other proteases are dependant on amino acid sequence and conformation near the cleavage site [6].

Rice tungro is a major rice disease in southeast asia and is caused by a double infection by two viruses [8,9]: Rice tungro bacilliform virus (RTBV) [10,11], a member of the genus *Badnavirus *in the family *Caulimoviridae *[1], and Rice tungro spherical virus (RTSV), a single-stranded RNA virus and member of the genus *Waikivirus *in the family *Sequiviridae *[12,13]. RTBV is responsible for symptoms of the disease [8] and RTSV is required for the transmission of the two viruses by the leafhopper vector *Nephotettix virescens *[14,15].

RTBV genome is a double stranded DNA of 8.0 kbp with two site-specific discontinuities resulting from replication by reverse transcriptase [3,16]. The RTBV genome has four open reading frames (ORF) [17]. ORF3 has similarities with the gag-pol core of retroviruses. The polyprotein (P3) contains a putative movement protein (MP), the capsid protein (CP), the aspartate protease (PR), and the reverse transcriptase (RT) with a ribonuclease H activity. The N- and C- terminal amino acid (aa) sequences of the CP were deduced from MALDI-TOF mass spectral analysis [18]. The location of the CP domain, which is encoded by aa 477–791 in P3, was confirmed by immunodetection reactions using antibodies raised against different segments of ORF3 [18]. P3 sequence aa 985–995 shows homologies to the active site of aspartate proteases encoded by retroviruses and pararetroviruses, with the sequence DSGS believed to be the RTBV protease active site. Changing of D to A (aspartic acid to an alanine) in this sequence affects the proteolytic processing of the RT [19]. Detection of RTBV gene products *in vivo *revealed a major protein of 13.5 kDa in virus preparations, by using an antibody raised against the domain aa 881–1098 [10,20]. The antibody was used in immunogold labelling reactions and showed binding on the surface of the virus particle, suggesting that the PR is located at or near the surface of the virus particle [20].

In this paper, we used mass spectrometry analysis to characterize the RTBV protease domain in a protein associated with purified virions, and confirm the hypothesis by *in silico *modelling and *in vitro *expression studies. Analysis of products of *in vitro *processing of P3 suggests that RTBV aspartate protease is involved in the release of the CP from the polyprotein P3.

## Results

### Determination of molecular mass

**Figure 1:**
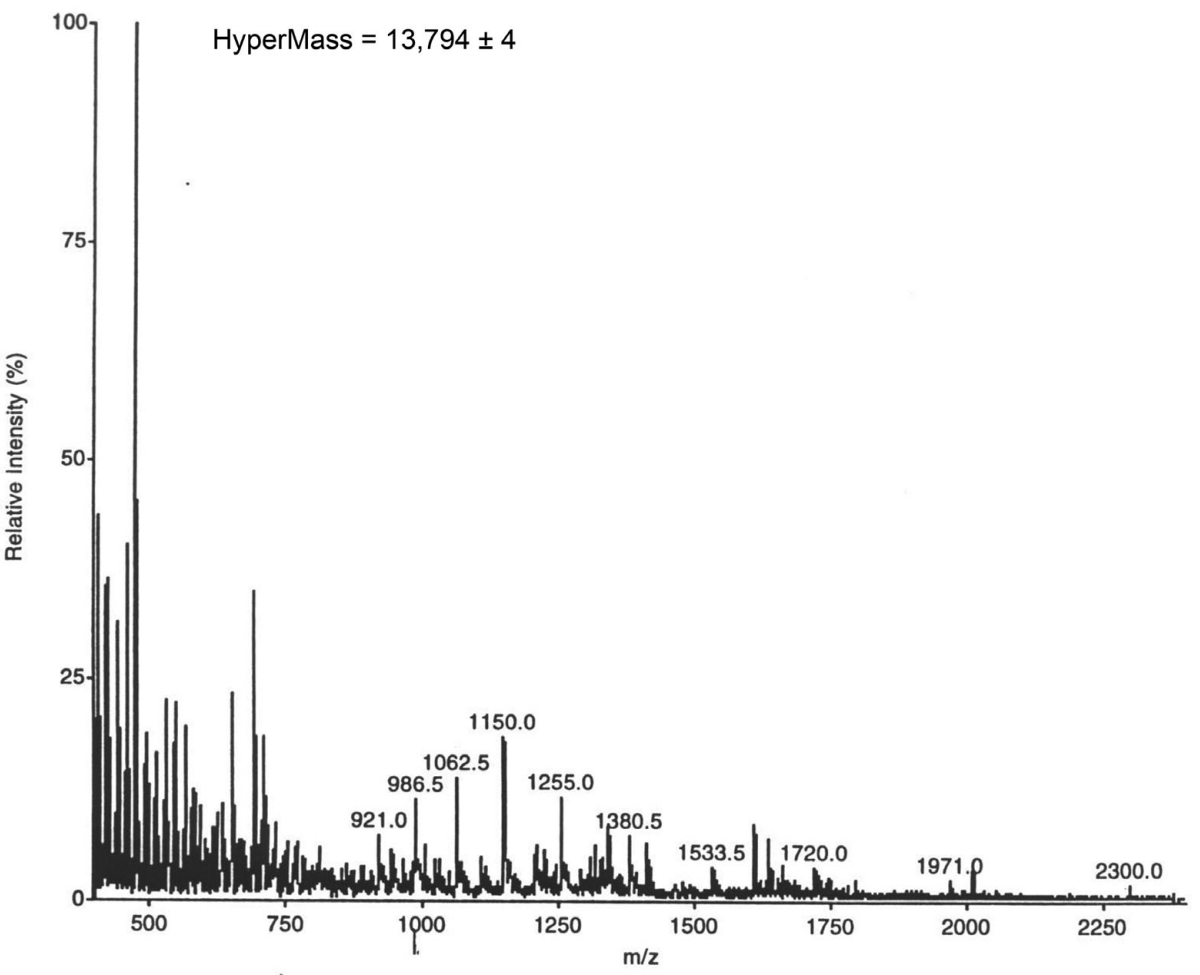
**Mass spectrometry analysis performed on RTBV virions**. In-line HPLC electrospray ionization mass spectrometry analysis performed on RTBV virions. Virus sample was denatured with guanidium hydrochloride 4.8 M prior, to injection onto the column. Analysis of peptide which coeluted from the in-line HPLC column in various charge states gave a molecular mass of 13,794 ± 4 Da.

In-line HPLC electrospray ionization was used to determine the molecular mass of proteins that were isolated in preparations of RTBV. Several peaks were observed using this method, none of which correspond to the known molecular mass of the CP subunits [18]. Many of the primary peaks were accompanied by low amounts of proteins of similar but not identical charge: we presumed that these peaks represented various charge statesof the peptide/protein. Reconstruction of the data, after algorithms were applied to convert the family of ion peaks, gave a protein with molecular mass of 13,794 ± 4 Da (Figure 1). The lack of recovery or detection of proteins of the size of CP subunits is likely the result of retention on the HPLC column due to the very basic charge of the protein; estimated isoelectric point (pI) of CP is 9.43.

### Localization of the protease domain

**Figure 2:**
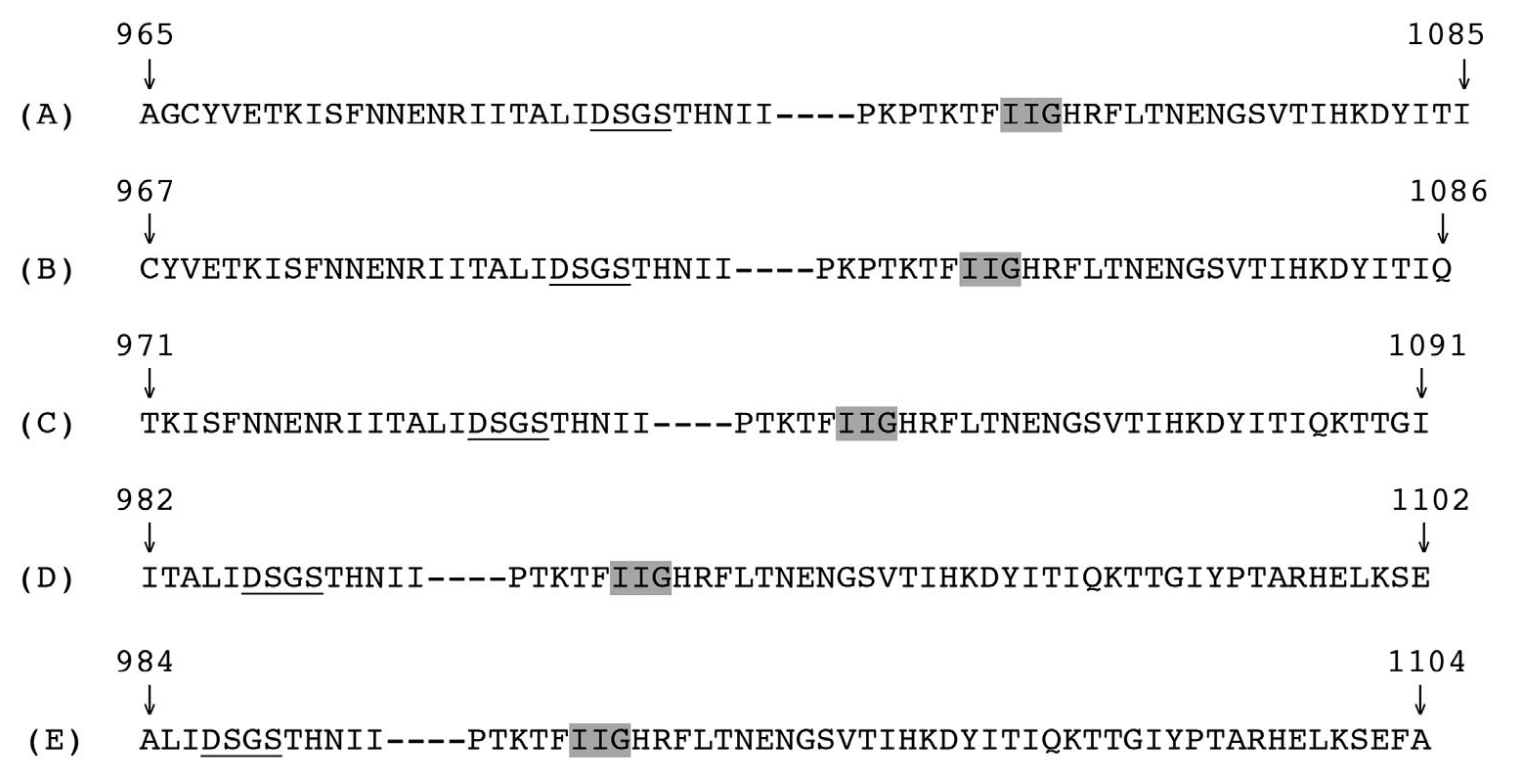
**Putative protease domains for RTBV**. Position of five peptides that include the active protease domains with molecular mass that correspond to the mass spectrometry analysis. Peptide A has a predicted molecular mass of 13,790.71 kDa; peptide B of 13,790.71 kDa; peptide C of 13,796.70 kDa; peptide D of 13,793.60 kDa; peptide E of 13,795.70 kDa. Underlined sequences represent the active site of the protease. The grey box indicates a conserved motif among retroviral proteases. Numbers above arrows indicate position of amino acids in P3.

By comparing the known sequence of P3 protein in RTBV with aspartate proteases encoded by other retroviruses we predicted that five possible proteins could be derived with a molecular mass of 13,794 ± 4 Da that could contain the putative active site domain DSGS (at aa 987–990) and the IIG sequence. The IIG sequence motif is conserved amongst retroviral proteases and is located at aa 1063–1065 in P3. The five predicted proteins (Figure 2) were covering amino acids 965–1085, 967–1086, 971–1091, 982–1102, and 984–1104, with predicted molecular masses of 13,790.71 Da, 13,790.71 Da, 13,796.7 Da, 13,791.7 Da and 13,795.7 Da, respectively. These predictions of the five domains are based on the combination of molecular mass and the presence of conserved motifs, and not on putative protease cleavage sites that flank the five predicted proteins.

### Structural model of the protease

**Figure 3:**
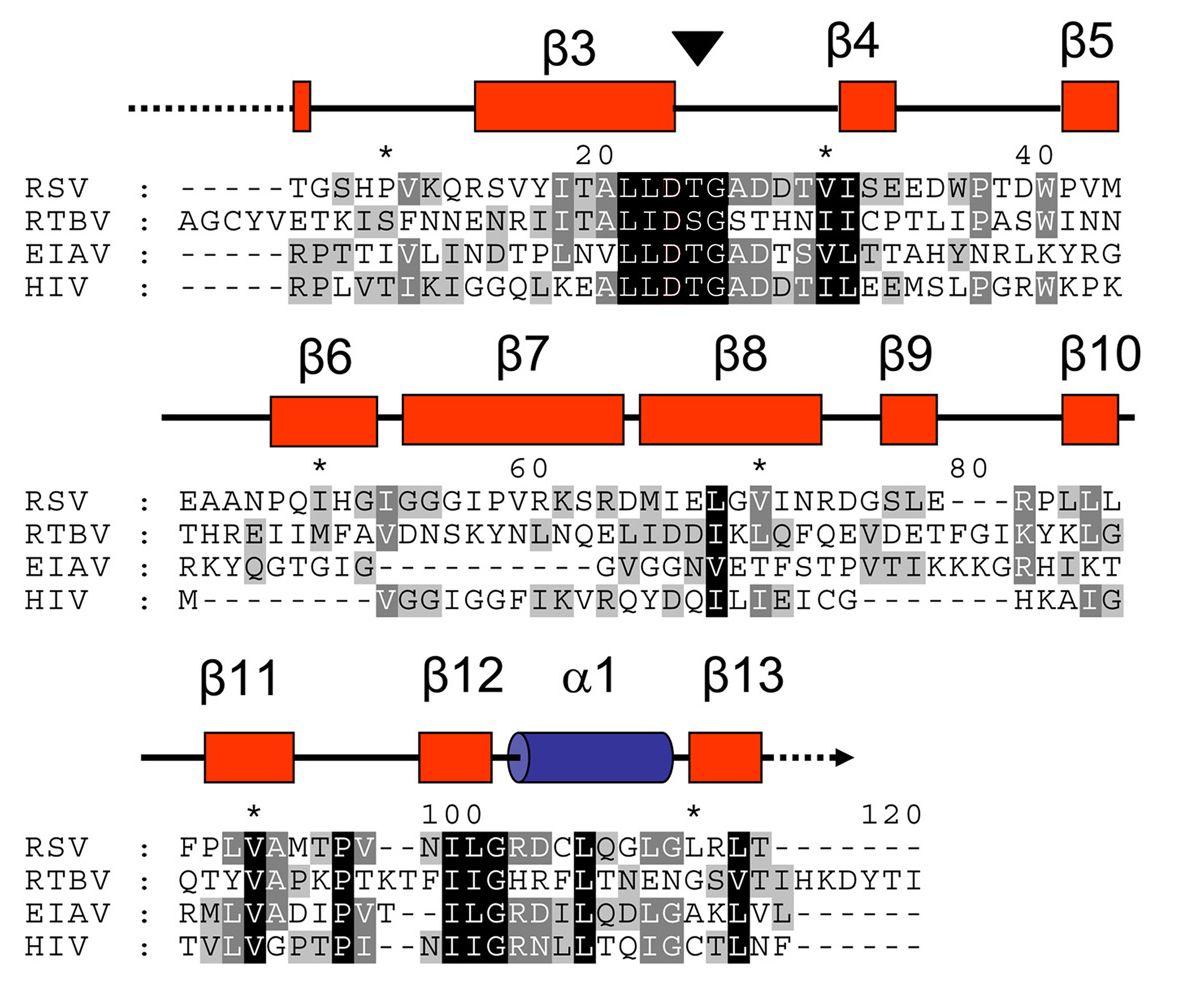
**Structural sequence alignment of the RTBV protease with other retroviral proteases**. Sequence alignment of the RTBV protease amino acid sequence with proteases of Rous sarcoma virus (RSV), Equine infectious anemia virus (EIAV) and Human immunodeficiency virus (HIV). The color scheme corresponds to percentage of similarity (based on physico-chemical properties). Black background and white foreground indicate 100%, grey background and white foreground indicate 80%, grey background and black foreground indicate 60%. Lower similarity values are not shown. Numbers over the alignment indicate the alignment length. Secondary structure elements from the RSV sequence are represented over the alignment. The numbering of the elements follows the RSV numbering based on structure. Boxes indicate beta strand elements assigned as β. The helix is represented as a cylinder and indicated as α. Thick lines connecting the elements are loops and dashed lines indicate a break in the sequence. The black triangle indicates the location of the active site.

**Figure 4:**
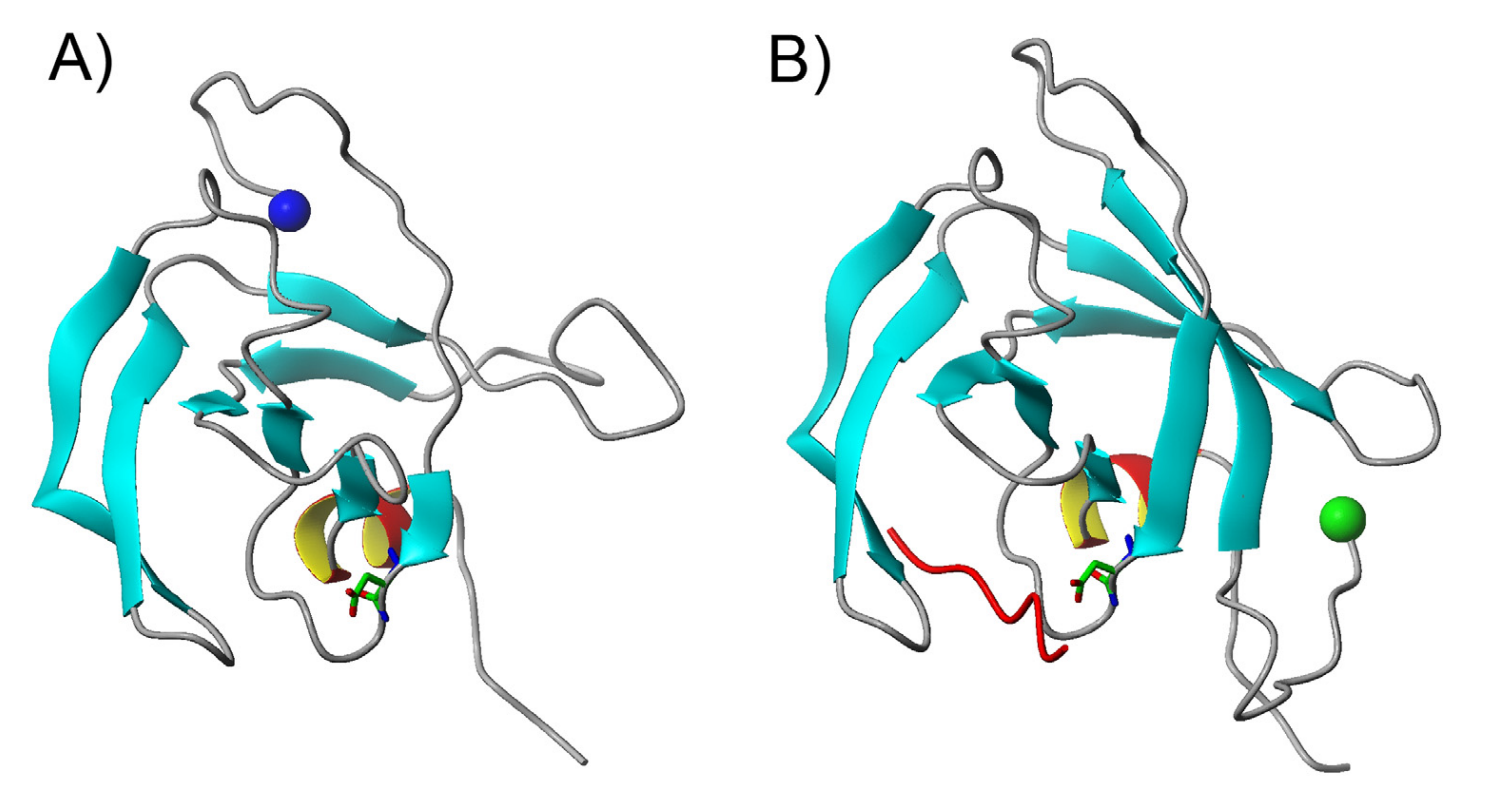
**Structural modelling of the RTBV protease**. Structural modelling of the RTBV protease (A), and Rous sarcoma virus (RSV) protease used as template (B). The sphere indicates the N-terminal end, aspartic acid of active site is shown in the stick model. In red is the RSV protease inhibitor 39 coupled to the active site. The first residues of RTBV PR could not be modelled. Conservation of the active site and overall fold recognition analyses with modelling building show that the RTBV sequence resembles greatly a protease fold.

The overall sequence similarity between aspartate proteases is quite low (below 25%) based on pairwise scoring criteria; therefore we applied fold recognition methods to detect remote homologues. The sequence alignment for the proteins selected for analysis showed strong conservation in the active site of the proteins (Figure 3). The pairwise alignment between the Rous sarcoma virus (RSV) template and the RTBV sequence was submitted to comparative modelling. As represented in the model (Figure 4), there is a strong resemblance between the structures predicted for RTBV and RSV proteases. The lack of identity in the predicted structures may be due to inherent inaccuracy of the modelling programs, due to inherent differences in the protein and substrate, and other characteristics. Taking these differences into account, as deduced by a folding assignment system [21] (where reliable scores were obtained) the protease sequence had a predicted folding structure that was highly similar to several other aspartate proteases. The overall fold surrounding the active site was well conserved between the putative RTBV protease and RSV protease used as template (Figures 3 and 4).

### Induction of putative protease domain

**Figure 5:**
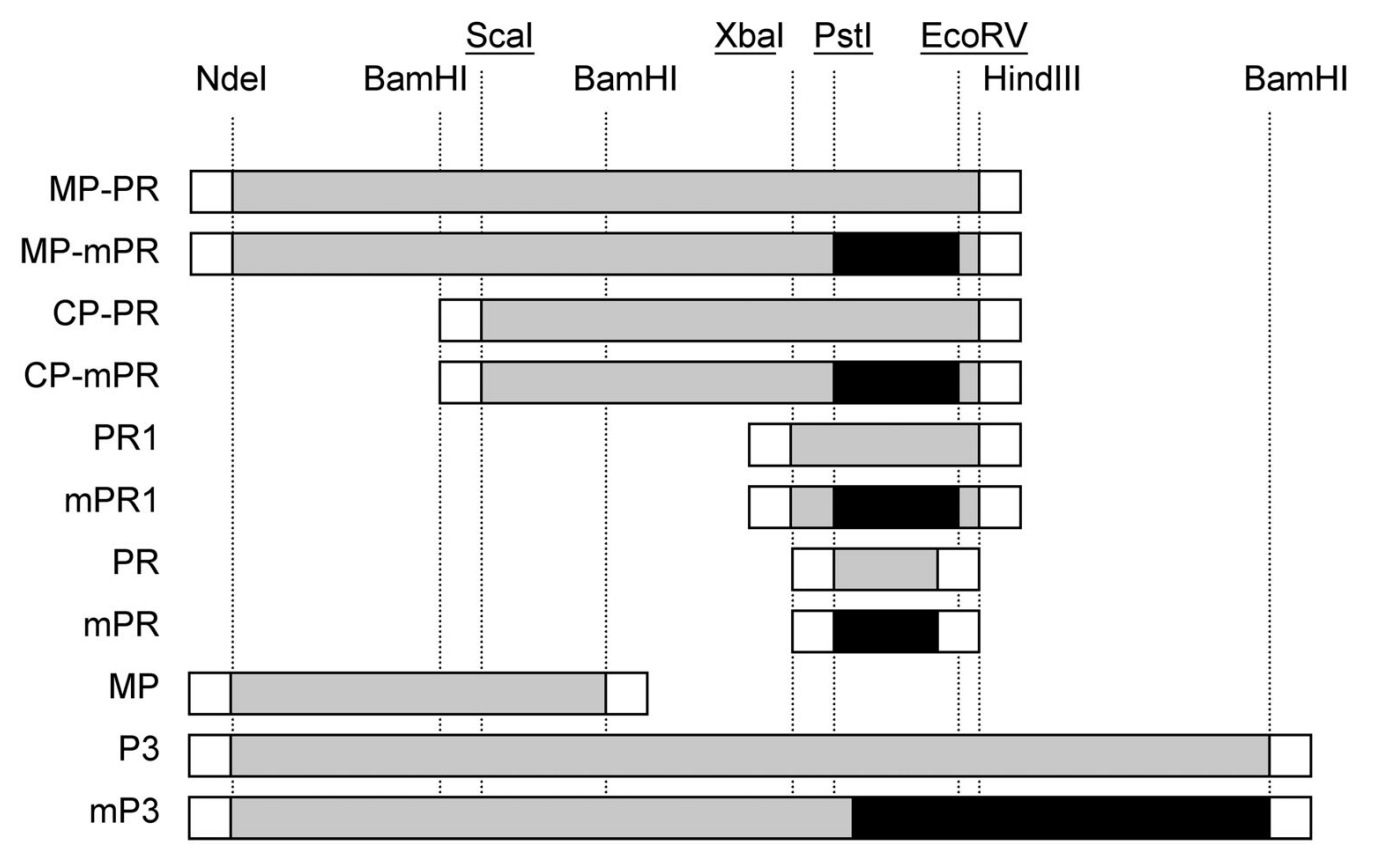
**Polyprotein P3 peptide domains cloned in different constructs**. Visualization of P3 peptide domains cloned in different constructs. Parts in grey are sequence derived from the full-length RTBV clone pBSR63A 11. Parts in black are sequence imported from plasmid pBS-mp/RT 19, containing the protease mutated active site. Parts in white are sequences from vectors. Underlined restriction enzymes are sites that are present in ORF3.

**Figure 6:**
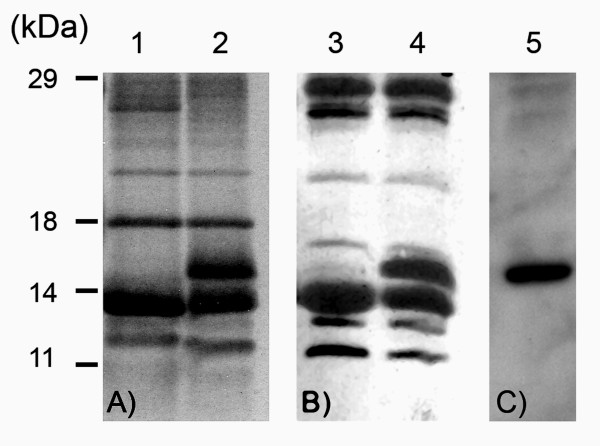
**Induction of the putative protease domain**. Expression of peptides in *E.coli*. Numbers on the left are estimated sizes in kDa of the molecular weight marker. (A) Coomassie blue-stained gel of induced peptides in *E.coli*. Lane 1: pTr-PR; Lane 2: pTr-mPR. (B) Western blot performed on induced peptides using antibodies raised against RTBV (Ab-RTBV). Lane 3: pTr-PR; Lane 4: pTr-mPR. (C) Western blot performed on induced peptides using antibodies raised against PR domain (Ab-PR). Lane 5: pTr-mPR. Peptide PR could not be induced from pTr-PR. pTr-mPR induced a specific peptide of about 14 kDa, corresponding to the protease domain (with mutation), and recognized by Ab-PR.

Specific primers (Ab-PR-F and Ab-PR-R) were designed to amplify the putative protease domain deduced from the mass spectrometry analysis (Figure 2-A). DNA used for PCR amplification included the full-length RTBV clone pBSR63A, and plasmid pBS-mp/PR; the reactions led to isolation of peptide PR and mPR, respectively (Table 1 and Figure 5). PR and mPR differ from each other only at amino acid 987, in which the residue was changed from Aspartic acid (D) to Alanine (A). Peptides were expressed in *E. coli *and, following induction of gene expression the extracts were subjected to SDS-PAGE, and gels were stained with coomassie blue (Figure 6-A). The expected size of the peptides was about 14 kDa. We did not detect a protein in cultures that contain pTr-PR. However, in extracts of cultures containing pTr-mPR, the protein mPR was easily detected at the expected position on the gel. Western immunoblot assays using antibodies raised against mPR (Ab-PR) confirmed that the 14 kDa peptide was mPR (Figure 6-C).

**Table 1:** Methodology for creating the constructs used in the analysis

*Cloning into pBlueScript KS*
Clone **pBS-CP/PR **was obtained by using primers CP-PR-F and CP-PR-R on full-length RTBV clone pBSR63A and cloning the PCR fragment into plasmid pBlueScript KS.
Clone **pBS-PR1 **was obtained by digesting pBS-CP/PR with *Xba*I and *Hind*III and cloning the 0.8 kbp fragment into plasmid pKS.
Clone **pBS-mPR1 **was obtained by digesting pBS-mpr/RT 19 with *Pst*I and *EcoR*V and cloning the 0.7 kbp fragment into pBS-PR1 digested with *Pst*I and *EcoR*V.
Clone **pBS-CP/mPR **was obtained by digesting pBS-mPR1 with *Pst*I and *EcoR*V and cloning the 0.7 kbp fragment into pBS-CP/PR digested with *Pst*I and *EcoR*V.

*Cloning into pTrHis*.
Clone **pTr-CP/PR **was obtained by digesting pBS-CP/PR with *BamH*I and *Hind*III and cloning the 2.5 kbp fragment into pTrHisA digested with *BamH*I and *Hind*III.
Clone **pTr-PR **was obtained was obtained by using primers Ab-PR-F and Ab-PR-Ron full-length RTBV clone pBSR63A and cloning the PCR fragment into pTrHis A digested with *Nco*I and *Hind*III.
Clone **pTr-mPR **was obtained by using primers Ab-PR-F and Ab-PR-R on plasmid pBS-mpr/RT and cloning the PCR fragment into pTrHis A digested with *Nco*I and *Hind*III.

*Cloning into pET*.
Clone **pET-MP **was obtained by using primers Et-MP-F and Et-MP-R on full-length RTBV clone pBSR63A and cloning the PCR fragment into pET-28a digested with NdeI and BamHI.
Clone **pET-MP/PR **was obtained by digesting pBS-CP/PR with *Sca*I and *Hind*III and cloning the 2.3 kb fragment into pET-MP digested with *Sca*I and *Hind*III.
Clone **pET-MP/mPR **was obtained by digesting pBS-CP/mPR with *Sac*I and *Hind*III and cloning the 2.3 fragment into pET-MP.
Clone **pET-P3 **was obtained by different steps. First, *Pst*I digested product (2.1 kbp) from pBS-PR/RT [19] containing PR and RT was cloned at the *Pst*I site of pTr-CP/PR. The resulting plasmid was digested by *BamH*I, and the 3.9 kb fragment was clone into pTrHis A, to obtain pTr-CP-RT. The later was digested with *Sca*I and *BamH*I and the 3.8 fragment was cloned into pET-MP digested with *Sca*I and *BamH*I to obtain pET-P3.
Clone **pET-mP3 **was obtained using the same strategy than above, by using pBS-mpr/RT instead of pBS-PR/RT.

*Lists of primers*
CP-PR-F : 5'-GAAAGAGGGATCCAAAATGGCAATAGTAGAAG-3'
CP-PR-R : 5'-GTTTTTCAAAAGCTTCTTAATCTGCTGGCGTG-3'
Ab-PR-F: 5'-CATGCCATGGCACATCATCATCATCATCATCATGCAGGATGTTATGTA-3'
Ab-PR-R : 5'-TATTCCCGAAGCTTTTTATATAGTTATATAATC-3'
Et-MP-F : 5'-GTAAGTGCCCATATGAGCCTTAGACCATTTACTGG-3'
Et-MP-R : 5'-AGGGCTGTGGGATCCTCATTCAGGTCTATCACCTC-3'

### Analysis of the processing of polyprotein P3

**Figure 7:**
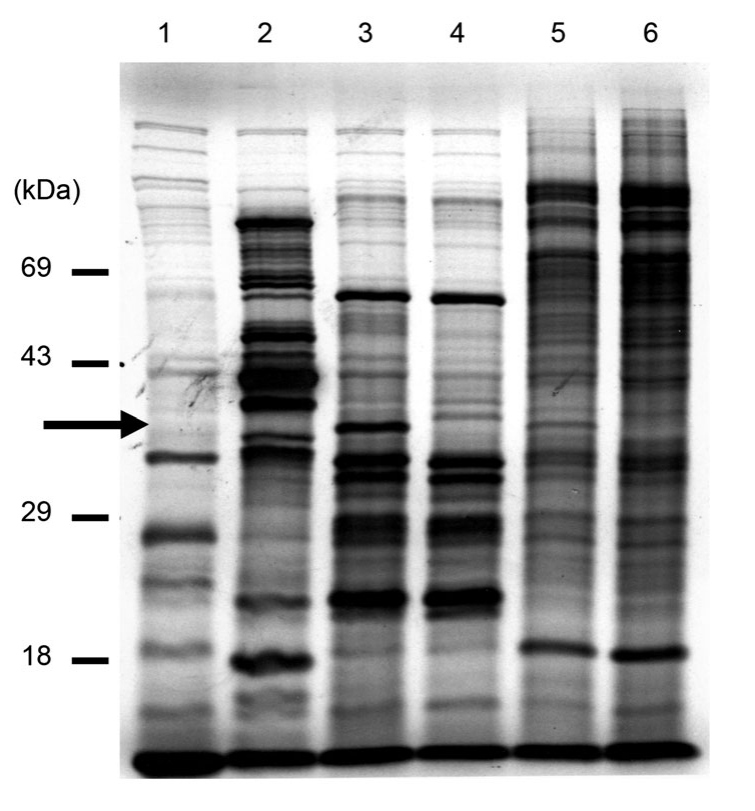
***In vitro *releasing of the coat protein from the polyprotein P3**. Autoradiography of an SDS-PAGE of induced peptides from different pET-vectors induced in *E. coli*. ^35^S radiolabelled methionine was added for 5 minutes after 60 minutes of induction with IPTG. Numbers (in kDa) on the left indicate mobility of the molecular weight markers. Lane 1: pET(no insert); Lane 2: pET-MP; Lane 3: pET-MP-PR; Lane 4: pET-MP-mPR; Lane 5: pET-P3; Lane 6: pET-mP3. Arrow shows the presence of a peptide (estimated molecular mass of 37 kDa) that is present only for constructs that code a peptide that contains the coat protein and the protease (pET-MP-PR; pET-P3).

Pulse-chase labelling techniques were used to reveal possible processing of P3 by the protease in *E. coli*. After 1 hour induction cultures were labelled with ^35^S-methionine for five min after which protein extracts were subjected to SDS-PAGE, followed by autoradiography. Multiple bands were observed in cultures that contain each plasmid (Figure 7). Protein patterns represent peptides that are expressed and processed between 60 and 65 min after induction. Patterns of peptides induced from construct pET-MP-PR and pET-MP-mPR were very similar with one visible difference, namely the presence of a band at 37 kDa in cells that contain pET-MP-PR. This band is absent in cells containing pET-MP-mPR, the plasmid with mutant PR. This difference was also observed between cultures that contain constructs pET-P3 and pET-mP3. The 37 kDa band was not present for the clone pET-MP and pET-28 (no insert) but was in cells that contain constructs that code a peptide that contains the CP and the protease, i.e. pET-MP-PRand pET-P3.

## Discussion

Retroviral proteases have been studied for many years as they are essential in the control of the replication of retroviruses. Retroviral proteases contain about 100 amino acids residues in length, and contain one copy of the active site Asp-Thr-Gly or Asp-Ser-Gly [22]. It was proposed that they are active as homo-dimers [23]. Crystallographic structures of Rous sarcoma virus (RSV) and Human immunodeficiency virus (HIV) showed that the PR of the viruses are dimeric, with two copies of the active site being brought into close proximity at the junction between the dimer partners [24,25]. To date, numerous retroviral proteases have been investigated, and their structures determined [26]. The retroviral protease is formed by duplication of four structural elements: a hairpin, a wide loop, an α-helix and a second hairpin. Active site sequences are placed in the extended loop of the structural model, implying that the active site is a number of amino acids away from the N-terminal of retroviral proteins.

The CP domain was previously characterized by MALDI-TOF (matrix-assisted laser desorption/ionization-time of flight) mass spectrometry analysis [18]. A single CP domain was identified, with positions of the amino- and carboxy- termini of the CP at aa 477 and 791, respectively. The molecular mass of the CP was determined to be 37,303 Da with an estimated pI of 9.43. A basic pI can explain the absence of peaks related to the CP in our mass spectral analysis. In this paper, virions of RTBV were subjected to in-line HPLC electrospray ionization mass spectrometry analysis. The HPLC column is expected to have retained the CP prior to mass spectrometry analysis. The molecular mass of the protein found by these analyses was determined to be 13,794 ± 4 Da, with a putative isoelectric point of 6.3. We suspected that this protein was the viral protease that was encoded in P3 protein.

Five peptide domains, with the predicted mass of the protein identified in virions, were found to contain the active site DSGS, the active site of an aspartate protease, and the conserved region IIG (Figure 2). We compared the derived sequences of the five putative proteins with sequences of retroviral proteases, a process that led us to predict that the peptide comprising aa 965–1085 represents the protease, based on the position of the active site within the predicted sequence. In the predicted protein the Aspartic acid residue of active site would be 23 amino acid away from N-terminus; the active site is 25 and 37 aa from the N-terminus for HIV and RSV, respectively.

To confirm if the target sequence is or is not a protease, we used a combination of sequence and structural predictions procedures to build a structural model for the RTBV protease (Figure 4-A) with RSV protease as a template (Figure 4-B). The consistency in fold recognition was given by reliable scores against different templates for the same SCOP classification (SCOP classification b.50.1). The proteins of this fold show a closed beta barrel. In the model, some of the beta strands elements are missing, however the remaining beta sheets can be arranged in a predicted conformation as they are product of sequence duplications. Keeping in mind that the protease domain is part of a multi-domain protein, additional interactions among the domains may influence the folding of individual subunits. The conservation in the predicted active site, and the predicted overall folding of sequences led us to predict that the domain posseses a protease activity. Subsequent experiment evidence that confirmed that the predicted active site can be inactive by mutation of D to A, the predicted secondary structure and the fold recognition analyses with model building led us to conclude that the protein comprises an aspartate protease.

Plasmids that encode peptides PR and mPR were introduced for expression in *E. coli*: however, PR did not accumulate in *E.coli *while mPR was expressed normally and reacted with Ab-RTBV and with Ab-PR antibodies. In other studies, the mutation of D to A in the active site of the RTBV protease was shown to affect its activity [19]. We suggest that PR did not accumulate in *E. coli *because the peptide was an active protease that was not tolerated in the host. Previous attempts to express proteases in *E. coli *have had similar outcomes [27,28].

**Figure 8:**
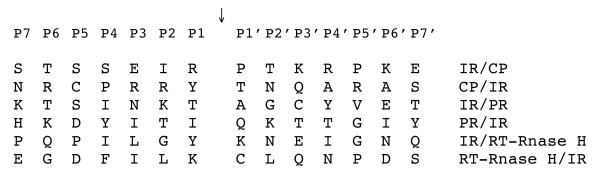
**Protease cleavage site sequences in the RTBV polyprotein P3**. Protease cleavage site sequences in the RTBV polyprotein P3. The designation of amino acid residues spanning the cleavage site is according to [40]. MP: movement protein; IR: intervening region; CP: capsid protein; PR: Protease; RT: Reverse transcriptase ; Rnase H: Ribonuclease H. Cleavage site sequences MP/IR has not been determined yet. A lack of significant sequence similarities is observed, a characteristic of aspartate proteases.

An antiserum against the region aa 881–1098 of the P3 was produced in previous studies [20]; this peptide includes the protease domain. Using this antibody a protein of approximately 13.5 kDa was detected by western blots assays in virus preparations and in infected tissues, suggesting that the protein that was detected represented the viral protease [20]. Furthermore, the antiserum was used to label virus particles, and revealed that the label was attached to virions. The characterization of PR domain, and the immunodetection reactions performed in the present studies are in agreement with the previous results, and also with studies performed by Marmey *et al*., [18], which investigated a peptide comprising aa 806–961 of P3, that was referred to as IR. IR did not react with Ab-RTBV serum, suggesting that the IR region did not contain sequences related to the CP. Our results support the hypothesis that peptide IR corresponds to the intervening region between CP and PR, and that it may be involved in the processing of P3 [18]. With the present study the N- and C- terminal amino acid sequences are now characterized for CP, PR and RT-Rnase H [18,19]. However, we did not identify apparent sequence similarities between the cleavage sites that would be used by the PR (Figure 8). Such a lack of sequence similarity is usual for viral aspartate proteases [29,30]. Other details that remain to beclarified in the organization of P3 include: characterization of the movement domain, and the order and rates in which the various sites on P3 are cleaved.

A previous work conducted in insect cells using baculovirus based constructs, including constructs in which the active site of the protease was mutated revealed that RT was processed by PR [19]. In the present work, *in vitro *processing of CP by PR was demonstrated in *E.coli*. If immunoprecipitations with antibodies were not achieved for technical reasons, presence of the 37 kDa peptide was associated with co-existence of CP and active PR in the construct. It is the first time that such a processing is demonstrated for pararetroviruses (e.g. *Commelina yellow mottle virus*, *Banana streak virus*, *Cacao swollen shoot virus*), where CP and PR are components of the same polyprotein.

Our results clarify the organization of P3, and its processing by its own protease and lead to a more complete understanding of the replication process and possible points of control of pararetroviruses.

## Methods

### RTBV strain used for the analysis

The RTBV strain used for the analysis was from the International Rice Research Institute (IRRI, Los Banos, Philippines). Sequence of the genome was published [11], with accession number [GenBank:M65026].

### Mutation of the active site of protease

Plasmid pBS-mp/RT [19] contain the putative mutated protease and reverse transcriptase of RTBV. The aspartic acid in the sequence DSGS (RTBV P3, amino acid 987) was changed to alanine, resulting in the sequence ASGS. Plasmid pBS-mp/RT was used for further sub-cloning.

### Analysis by mass spectrometry

In-line HPLC electrospray ionization mass spectrometry [31] was employed. The experimental protocol was similar to that described [19]. MacBioSpec algorithms (Sciex) were used to convert the family of ion peaks, which result from the protein being in various charge states, to an accurate molecular mass. Virus sample was denatured with guanidium hydrochloride at 4.8 M prior to injection onto the column.

### Sequence and structural prediction analyses

The RTBV sequence was used as a query for the BLAST program against non redundant databases and PDB databases. No significant hits were identified with suitable e-values by these queries. The sequence was then submitted to fold recognition methods at the metaserver [32]. Reliable templates were found with high scores, all of which were found in retroviral proteases (all beta proteins: SCOP classification b.50.1.1). Several pairwise alignments between RTBV and templates were checked using SQUARE [33] and further submitted to homology modelling using the Swissmodel program [34]. Models were evaluated using PSQS [35] and Whatif [36] tools. The structure of the Rous sarcoma virus (RSV) protease (pdb code 1bai_A) provided the best model as a viral aspartate protease and was chosen for this purpose. Illustrations for the model were generated using MolMol [37].

### Constructions of plasmids

The full-length RTBV clone pBSR63A [11] was used as DNA matrix for PCR reactions to amplify specific regions of ORF3 using specific primers that were designed to amplify specific sequences from the RTBV genome. Constructs were obtained by cloning the PCR fragments into vectors and/or by cloning fragments obtained after digestion of constructs with restriction enzymes and pBS-mp/PR (Table 1). Cloning was conducted in pBluescript KS vector (Stratagene/USA), in pTrHis vector (Invitrogen/USA) and in pET vector (Novagen/USA). Restriction enzymes were used according to manufacturer (Gibco-BRL, USA).

### Expression of proteins

All pTrHis based vectors were transformed into *E.coli *DH5-α. The resulting plasmids were designated pTr-PR, pTr-mPR and resulted in synthesis of peptides, named PR, mPR, corresponding to regions between residues 965–1085 (Figure 5), with plasmid pTr-mPR encoding peptide with amino acid 987 mutated from D to A.

### Analysis of *in vitro *processing

All pET vectors were transformed into *E.coli *BL21/DE3 (pLys S). The resulting plasmids were designated pET-MP, pET-MP/PR, pET-P3, pET-MP/mPR, pET-mP3. These plasmids encode (in order) peptides, named MP, MP-PR, P3, MP-mPR, mP3 corresponding to regions between residues 1–606, 1–1195, 1–1675, 1–1195, 1–1675, respectively (Figure 5). Plasmids pET-MP/mPR and pET mORF3 encode peptides with amino acid 987 mutated from D to A. Bacteria were induced with IPTG in M9 salts medium, using rifampicin and an amino acid mixture that lacks methionine. At 60 minutes, ^35^S radiolabelled methionine was added for five minutes. Bacteria were centrifuged for three minutes and resuspended in Laemli sample buffer [38]. Samples were subjected to electrophoresis, the gel was dried and exposed to x-ray film overnight.

### Antibodies and Western blot analysis

Antibodies were obtained as previously described [18]. Peptide mPR, corresponding to region between residues 965–1085 and expressed with construct pTr-mPR, was used to produce Ab-PR. An antiserum (Ab-RTBV) was also raised against purified virions. Proteins were subjected to electrophoresis in SDS/PAGE, and transferred to a nitrocellulose membrane. The blot was incubated with antiserum at a 1:1000 dilution. Immunogenic proteins were detected using an alkaline phosphatase goat anti-rabbit IgG at 1:10000 dilution. Proteins were visualized in the presence of nitroblue tetrazolium and 5-bromo-4-chloro-3-indoyl phosphate, or using the Biomax chemiluminescent detection system (Kodak/USA).

## Competing interests

The author(s) declare that they have no competing interests.

## Authors' contributions

PM carried out the designing of primers, the construction of various plasmids, the *in vitro *expression analysis and pulse chase labelling experiments; ARM performed the structural modelling analysis; AdK analyzed various sequences by computer; RNB and CMF were principal investigators. All authors read and approved the final manuscript.
